# Galvanic vestibular stimulation reveals disruption of ipsilesional brainstem pathways in hemiparetic stroke survivors

**DOI:** 10.1113/EP093389

**Published:** 2026-05-22

**Authors:** Angelo Bartsch‐Jiménez, Hesam Azadjou, Francisco J. Valero‐Cuevas

**Affiliations:** ^1^ Division of Biokinesiology and Physical Therapy University of Southern California Los Angeles California USA; ^2^ Escuela de Kinesiología, Facultad de Medicina Universidad de Valparaíso Valparaíso Chile; ^3^ Alfred E. Mann Department of Biomedical Engineering University of Southern California Los Angeles California USA

**Keywords:** galvanic vestibular stimulation, stroke, vestibular output, voluntary reaching

## Abstract

The spatiotemporal structure of muscle coordination emerges from the collaboration and competition among cortical, brainstem and spinal pathways onto motor neuron pools, each continuously shaped by task demands, limb position and descending tract integrity. Here, we used galvanic vestibular stimulation (GVS) to investigate whether brainstem vestibular output is disrupted in stroke survivors with right hemiparesis (*n* = 14) compared with age‐matched control subjects (*n* = 14). We estimated this via intermuscular coherence among neck and arm muscles during both rest and a reaching‐like (i.e., reaching) movement with the shoulder in neutral and abducted positions, each with no stimulation, sham stimulation or GVS. Previous work showed that young adults exhibit increased coherence between neck muscles with GVS at rest, as do our control subjects. Surprisingly, we saw the same pattern in stroke survivors only on their paretic side, with reduced coherence in their non‐paretic side. During reaching, the paretic side did not show changes in coherence between arm or neck muscles, in comparison to control subjects, with and without GVS, and importantly, even lower coherence with the abducted shoulder. Given that GVS did not exacerbate intermuscular coherence in any muscle pair during the reaching tasks and that coherence was even reduced during shoulder abduction, our findings provide evidence to exclude increased brainstem vestibular output as a dominant contributor to pathological synergies during voluntary movement following stroke. In addition, the decreased coherence between neck muscles of the non‐paretic side during GVS at rest suggests changes in ipsilesional brainstem vestibular output. This highlights opportunities to consider unexplored contralesional brainstem–spinal pathways, in addition to downregulated ipsilesional vestibular projections, in neurorehabilitation strategies following stroke.

## Abstract (highlights)

- **What is the central question of this study?**
  Pathological synergies following stroke are linked to corticospinal injury and increased subcortical drive; however, it is unclear whether vestibular output is also disrupted, especially during voluntary reaching movements.
- **What is the main finding and its importance?**
  Stroke survivors did not show increased vestibular drive to arm muscles during reaching movements with either a neutral or abducted shoulder. Importantly, galvanic vestibular stimulation did not increase vestibular drive to arm muscles at rest or during reaching tasks. However, stroke survivors showed decreased vestibular drive to non‐paretic neck muscles. These findings argue against increased vestibular output as a contributor to pathological synergies but highlight ipsilesional brainstem pathways as targets for rehabilitation.
- **Key points**
  - Muscle coordination emerges from the interaction of cortical, brainstem and spinal pathways.
  - We used galvanic vestibular stimulation (GVS) to assess whether brainstem vestibular output is disrupted in stroke survivors.
  - At rest, GVS increased intermuscular coherence of paretic but not non‐paretic neck muscles in stroke survivors.
  - During reaching, intermuscular coherence of paretic arm muscles was reduced, especially when the shoulder was abducted. However, there was no effect when GVS was applied.
  - These findings suggest disrupted vestibular output to the non‐paretic arm but argue against its increased drive as a dominant contributor to pathological synergies.

## 1 INTRODUCTION

Motor impairment following stroke includes disruptions to arm and hand movements by exhibiting ‘pathological’ synergies, which reflects reduction in the independent control of muscles. The flexion synergy of the paretic arm is characterized by abnormal coupling of shoulder abduction, elbow flexion and forearm supination (Brunnstrom, 1970; Dewald et al., 1995; Twitchell, 1951). Importantly, this abnormal muscle coupling is exacerbated during shoulder abduction (Dewald et al., 1995; McPherson et al., 2018). This results in impaired voluntary reaching and grasping movements, including a reduced ability to control hand opening and grasping forces (Lan et al., 2017), reduced elbow and shoulder excursion angles (Beer et al., 2007), and decreased reaching velocity, distance and working area (Ellis et al., 2018; McPherson et al., 2018; Sukal et al., 2007).

The proposed primary mechanism underlying pathological synergies is the disruption of corticospinal tract (CST) drive, compromising monosynaptic corticomotoneuronal projections to muscles. Two additional key mechanisms are likely to contribute to its emergence: (1) increased drive from descending subcortical (brainstem) pathways; and (2) maladaptive changes in segmental reflex circuits (Krakauer, 2005). Motor signs arising from decreased drive following CST disruptions are commonly classified as negative signs, including weakness (paresis or plegia) and dexterity loss (fractionated motor control). In contrast, positive signs include hyperreflexia, increased muscle tone (spasticity), abnormal resting postures and pathological synergies (Hadjiosif et al., 2021, 2022; Kamper et al., 2006; Krakauer, 2005). Although the underlying circuitry of the CNS is highly complex, and multiple interactions among mechanisms are likely, it has been proposed that positive signs arise following loss of supraspinal and cortical inhibitory modulation of subcortical motor pathways (Huang et al., 2024; Li & Francisco, 2015; Mukherjee & Chakravarty, 2010). The reticulospinal tract has been proposed as one contributor to pathological synergies (positive sign), either by increased drive and/or by maladaptive reflex changes. For example, stroke survivors have shown increased reticulospinal tract drive to ipsilateral arm muscles when triggering StartReact responses (Choudhury et al., 2019), and during transcranial magnetic stimulation alone or paired with startle stimulation (Mooney et al., 2025; Taga et al., 2021). Likewise, the paretic limb of stroke survivors shows increased reticulospinal tract recruitment, which is correlated with both shoulder abduction loading and expression of synergy (McPherson et al., 2018). The vestibular system is another possible contributor to motor impairment following stroke. The vestibular system is thought to regulate the excitability of alpha and gamma motoneurons and to modulate stretch reflex amplitudes (Lance & McLeod, 1981; Molina‐Negro et al., 1980).

Vestibulospinal reflexes play an important role in activating axial and limb musculature. These reflexes, such as the vestibulocollic reflex, are mediated by the medial vestibulospinal tract, but consist of indirect pathways that include spinal cord interneurons and reticulospinal pathways (Cullen, 2023; Urbin, 2025). The medial vestibulospinal tract has been studied by using ipsilateral galvanic vestibular stimulation (GVS), which activates vestibular afferents through current applied via surface electrodes on the mastoid process (Cullen, 2023; Kwan et al., 2019).

It has been shown that such activity is regulated by voluntary motor commands, such as when common neural drive to neck muscles is suppressed during voluntary arm movement (Bartsch & Valero‐Cuevas, 2025). Moreover, in the upper extremity, muscle responses to vestibular stimulation (assessed by vestibular evoked myogenic potentials) have been recorded in the deltoid, biceps, triceps, brachioradialis, flexor carpi radialis and palmaris longus (Cherchi et al., 2009; Papathanasiou et al., 2013; Valente et al., 2020). Following stroke, vestibular‐evoked myogenic potentials show an asymmetric behaviour, increasing amplitudes on the paretic side compared with the non‐paretic side. Importantly, they are strongly correlated with spasticity (Miller & Rymer, 2017; Miller et al., 2014, 2016). These findings provide evidence not only of altered brainstem vestibular output to neck and arm muscles after stroke, but also of maladaptive changes in reflex responses probably arising from upregulated vestibular drive.

Although the role of the reticulospinal tract in pathological synergies has been relatively well studied, the vestibular contribution to pathological synergies remains understudied. In particular, the asymmetric responses to vestibular stimulation observed in stroke survivors might reflect either an upregulation of vestibular drive or an increase in common drive to muscles on the paretic side, potentially contributing to pathological synergies. The medial and lateral vestibulospinal tracts have bilateral and ipsilateral projections, respectively (Cleland & Madhavan, 2021; Lemon, 2008). Consequently, both vestibular pathways from the contralesional brain project to the paretic side of stroke survivors. In addition, pathological synergies are exacerbated during reaching tasks that involve shoulder abduction. Thus, it is also crucial to determine whether vestibular output to arm muscles is increased in a similar manner during reaching with the abducted shoulder. To probe the potential contribution of a vestibular mechanism to impairment of the paretic arms, the aims of this study are as follows: (1) to assess brainstem vestibular output to shared neural drive, as measured by intermuscular coherence (IMC), at rest and during voluntary reaching movements in the paretic and non‐paretic arms of stroke survivors; and (2) to establish whether IMC increases with GVS and shoulder abduction. Ultimately, our findings will refine our understanding of the interplay between cortical and subcortical pathways on both sides of the brain in hemiparetic stroke, offering new insights into the neural origins of pathological synergies, which will be useful for developing targeted neuromodulatory and neurorehabilitation strategies.

## 2 MATERIALS AND METHODS

### 2.1 Ethical approval

The study conformed to the standards set by the *Declaration of Helsinki*, except for registration in a database. All participants gave their informed written consent to participate in this study, which was approved by the University of Southern California Internal Review Board (HS‐19‐00062).

### 2.2 Study participants

Fourteen chronic stroke survivors who were right‐handed prior to stroke participated in the study (*n* = 14; nine males and five females), with a mean age of 58.5 ± 8 years. Time since stroke onset was 4.6 years on average, ranging from 1.1 to 14 years (see Table 1). Inclusion criteria included a single unilateral cortical or subcortical brain lesion resulting in right hemiparesis. Participants were recruited across a wide range of upper extremity impairment scores (Fugl‐Meyer score range, 6–60; mean, 40.9 *± *19.6) to reflect the clinical spectrum of chronic hemiparetic stroke and maximize generalizability. Despite variability in time since stroke (1.1–14.1 years), the neuroplasticity window is considered closed at the chronic stage, and substantial recovery is no longer expected (Murphy & Corbett, 2009; Savitz, 2022). Importantly, participants with mild‐to‐moderate impairment continue to exhibit pathological synergies, particularly under shoulder abduction load (Dewald et al., 1995; McPherson et al., 2018). Consequently, the inclusion of a shoulder abduction condition ensured that synergy‐related muscle coupling was elicited experimentally across participants, independently of their resting Fugl‐Meyer score or time since stroke onset. A trained physical therapist assessed upper extremity function using the 66‐point upper extremity motor domain of the Fugl‐Meyer assessment (Fugel‐Meyer et al., 1975). The Fugl‐Meyer score ranges from 0 to 66, where a maximal score indicates normal motor function. Additionally, all participants self‐reported no history of vestibular, neurological or auditory deficits. An age‐matched group was composed of 14 right‐handed participants (*n* = 14; 10 males and 4 females), with a mean age of 58.3 ± 8.6 years. Importantly, all participants in the control group were free from any neurological condition affecting control of the upper extremity (neurotypical).

Stroke survivors had a stroke in their left hemisphere, resulting in right‐side hemiparesis. The labels paretic and non‐paretic pertain to vestibular structures (i.e., otolith organs and semicircular canals) and muscles of the neck and arm on the corresponding side of stroke participants. GVS increases vestibular drive ipsilateral to the positive electrode location, and the results are always described ipsilateral to the paretic‐centred frame of reference (for a description of the GVS protocol, see subsection 2.4). For example, when describing the effect of GVS on the paretic side, the anode (positive electrode) is located on the right mastoid process, and we measured muscle activity from the same (paretic) right side. Finally, to avoid confusion, we refer to sides as affected, (ipsi)lesional, contralesional or contralateral only if necessary.

### 2.3 Tasks

Participants performed the following tasks while sitting.

**FIGURE 1:**
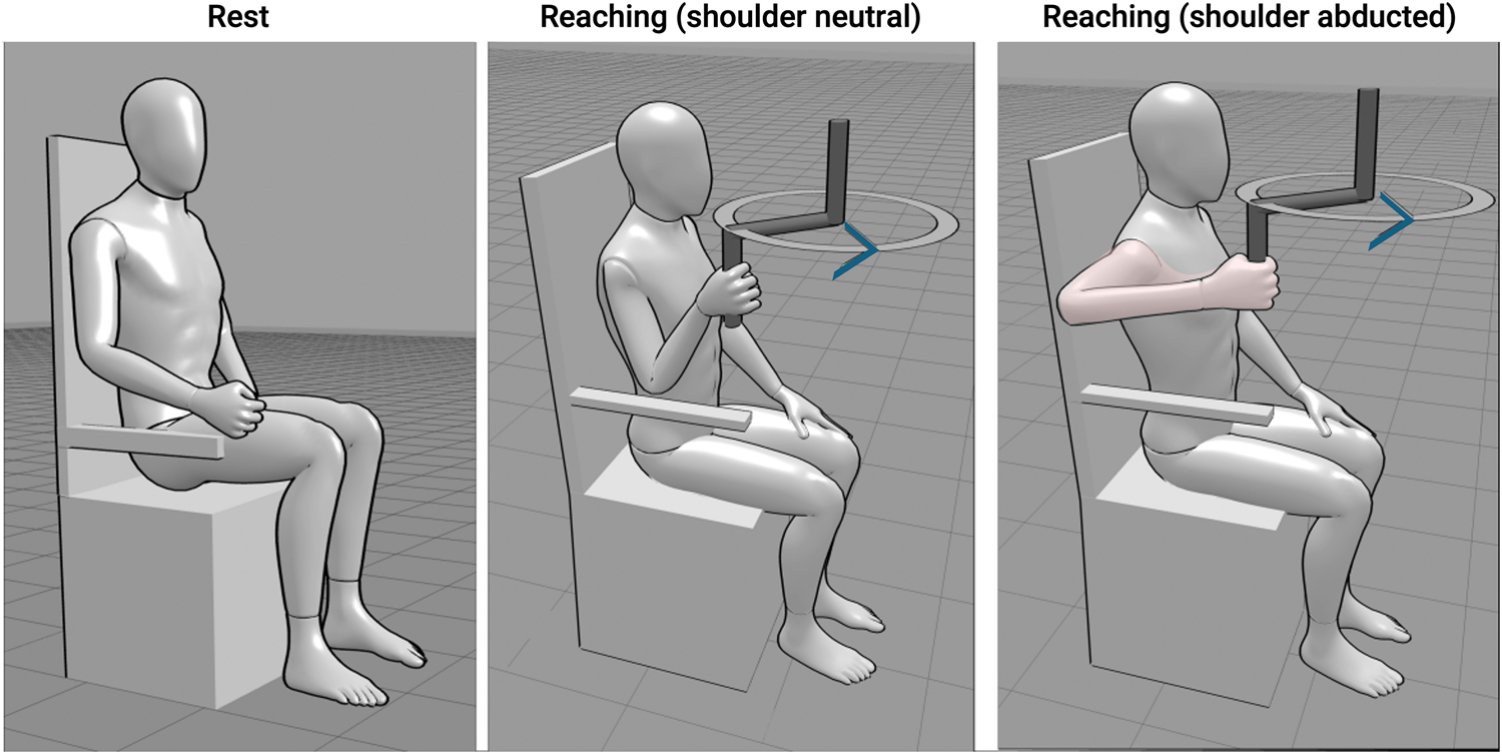
Tasks performed by each participant. The left panel shows that at the beginning of the experiment, each participant sat comfortably in a chair with their hands on their lap, while EMG was recorded at rest. The middle panel shows that during the reaching task, participants are asked to rotate a horizontal ergometer inwards (anticlockwise for the right arm, and clockwise for the left arm) to produce a cyclical movement. The right panel shows that, in the reaching with abducted shoulder condition, subjects were asked to perform the same reaching‐like movement while keeping their shoulder abducted at 90°. Verbal encouragement was constantly provided to avoid shoulder drifting.

**Rest**: Participants were seated with their hands resting on their lap or armrest. They were encouraged to stay relaxed and silent for 90 s at the beginning of the experimental procedures to collect baseline muscle activity (Figure 1, left panel).

**Reaching (neutral shoulder position)**: Participants were seated in front of a hand‐powered ergometer mounted to be rotated in the horizontal plane with each arm separately (Figure 1, middle panel). The ergometer provides a stable and standardized cyclical reaching‐like movement pattern, while also ensuring a mirrored movement between arms. The protocol for this task has been described thoroughly in previous articles (Bartsch & Valero‐Cuevas, 2025; Erwin et al., 2026; Laine et al., 2021). In summary, the movement direction was mirrored between arms, with an anticlockwise rotation when it was performed with the right or paretic arm and clockwise when performed with the left or non‐paretic arm.

**Reaching with abducted shoulder**: Participants were seated and performed the same movement as in the previously described task, but with a constant shoulder abduction of 90° (Figure 1, right panel).

**FIGURE 2:**
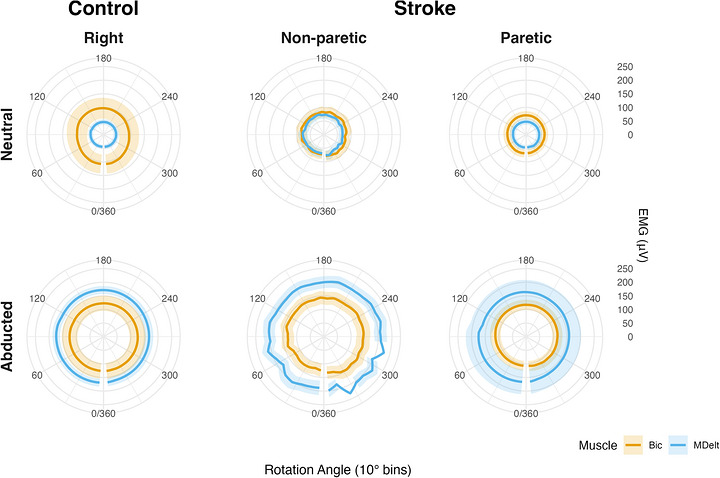
Polar plot of group‐averaged EMG across the reaching cycle (10° bins) for middle deltoid (MDelt) and biceps brachii (Bic) in the right arm of control participants and in both arms of stroke participants with neutral versus abducted shoulder positions. An a priori analysis (paired SPM*{*t*}*, Abducted *> *Neutral) revealed significantly increased activity in the abducted shoulder condition for both muscles. Note that the non‐paretic arm (left) rotated anticlockwise during these tasks.

Verbal encouragement and feedback were given to help the subjects to maintain the instructed position if they deviated. Rest breaks were allowed in the event of fatigue, but no participant requested them. During the reaching task with the abducted shoulder, 11 participants from the stroke group were able to finish the task successfully with their paretic arm. Consequently, the data from three participants were not included in the final analysis owing to inability to complete the task with abducted shoulder (two participants) or owing to technical difficulties during the experimental procedure for one participant (Table 1). All participants from both groups successfully completed all the remaining tasks with both arms, and their data were included in the statistical analyses to maximize statistical power. To determine whether the abducted shoulder position increases muscle activity in the biceps and middle deltoid muscles, we performed an a priori comparison between the neutral and abducted conditions for each muscle and each side of the stroke group independently. To ensure that individual reaching movements were comparable despite natural variability in movement duration and speed, each rotation was cycle normalized. This procedure rescales the time axis of every movement to a common 0°–360° cycle, allowing corresponding movement phases to be aligned across repetitions and participants. After normalization, EMG signals were averaged within 10° bins across the reaching cycle and compared between conditions using paired statistical parametric mapping. This analysis revealed significantly higher muscle activity in the abducted shoulder position relative to the neutral position for the biceps and middle deltoid muscles (Figure 2).

### 2.4 Stimulus types

**FIGURE 3:**
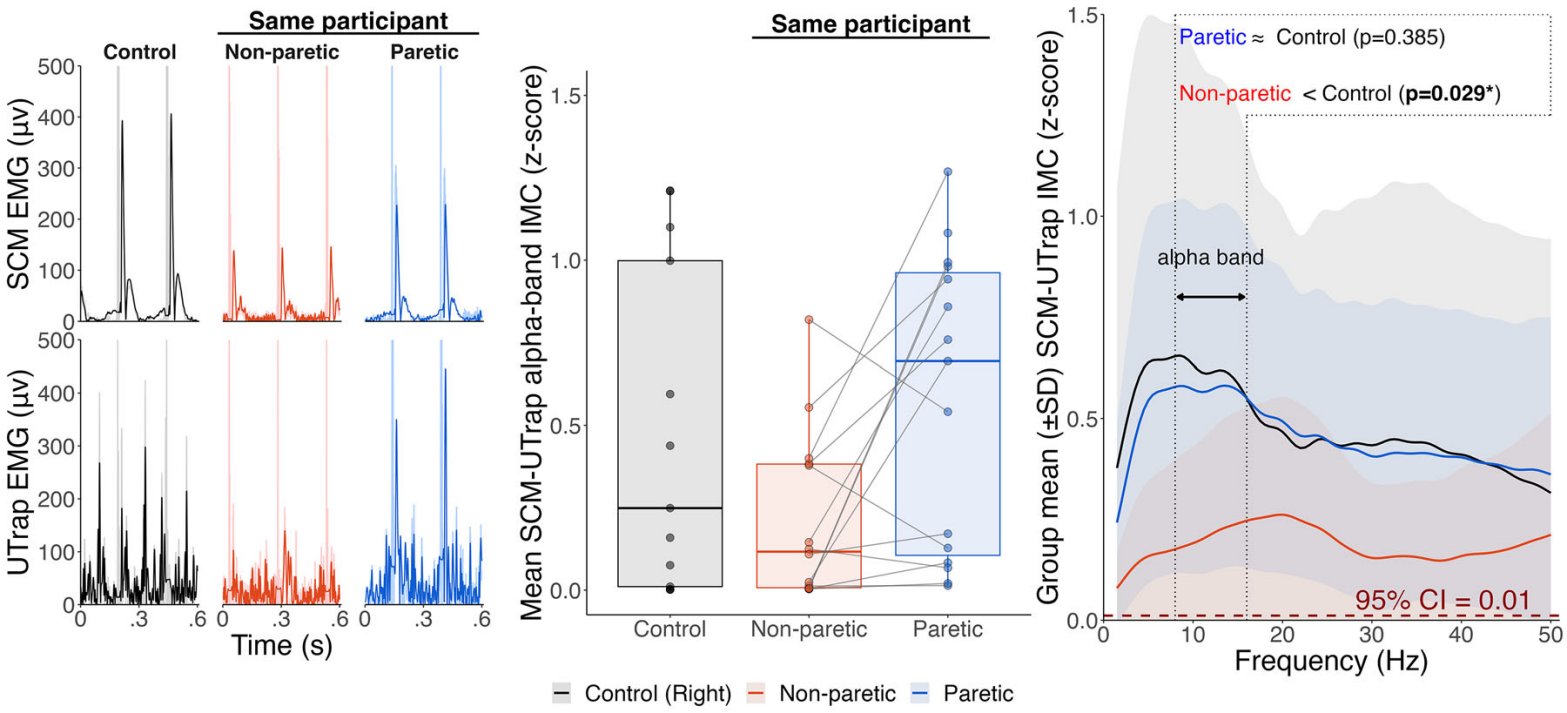
At rest, EMG signals and intermuscular coherence *z*‐score (IMC*_z_*) reveal lower responses to galvanic vestibular stimulation (GVS) in the non‐paretic side (red trace) of stroke survivors, compared with the right side of control participants (black trace). The left panel shows the raw (light trace) and processed (dark trace) EMG signals for the sternocleidomastoid (SCM) and upper trapezius (UTrap) muscles in a representative participant. Note the lower GVS‐driven responses on the non‐paretic side of both muscles. The middle panel shows a boxplot with individual IMC*_z_* means in the alpha band, highlighting the lower coherence levels on the non‐paretic side, compared with the paretic side and with control participants. The right panel shows the group mean IMC*_z_* (±SD) for the SCM–UTrap muscle pair. Significant differences in the alpha band (8–16 Hz) were observed between the non‐paretic side and control participants (*P* = 0.029), but no significant differences were found between the paretic side (blue trace) and control participants (*P* = 0.385). Values above the dashed dark red line can be considered to have significant IMC*_z_*, according to the 95% upper confidence interval estimated from 1000 randomizations of the original signals.

During each task, we applied three stimulus types: no stimulation, GVS, and sham stimulation (for a detailed description, see Bartsch & Valero‐Cuevas, 2025). GVS activates afferents from both the otoliths and semicircular canals, eliciting oculomotor and postural responses (Curthoys & MacDougall, 2012; Forbes et al., 2015; Marchand et al., 2025). Consequently, the body sways and the gaze shifts towards the anode, with a task‐dependent increase in activity in the postural muscles (Bartsch & Valero‐Cuevas, 2025; Forbes et al., 2015; Marchand et al., 2025). Based on the facilitatory effects observed in lower extremity postural muscles under analogous electrode configurations (Bartsch & Valero‐Cuevas, 2025; Forbes et al., 2015), we hypothesized that GVS would increase muscle activity ipsilateral to the anode, reflecting increased vestibulospinal drive to the tested arm, and thereby modulate intermuscular coherence during voluntary tasks. The selected GVS protocol consisted of a binaural galvanic stimulation, where the anode (positive electrode) was placed to induce a vestibular response (on the tested side), which was verified by visual inspection of the sternocleidomastoid (SCM) muscle (Forbes et al., 2015). The stimulation frequency was set at 4 Hz, with an amplitude ranging between 0.8 and 1.2 mA and a pulse duration of 2 ms (Bartsch & Valero‐Cuevas, 2025; Kwan et al., 2019). To prevent artefactual coherence within the alpha‐to‐gamma frequency bands (8–50 Hz), we used a stimulation frequency of 4 Hz. The stimulation amplitude was set for each participant at the beginning of the session to avoid EMG signal saturation from the SCM muscle or if the participant felt uncomfortable. Participants were instructed and encouraged to report verbally any pain or dizziness arising from GVS. Only one participant reported pain before the minimum amplitude of 0.8 mA was reached; their session concluded before any data were collected. No other participants reported dizziness, pain or discomfort. After reaching a comfortable amplitude (within the 0.8–1.2 mA range), each participant received the same amplitude on both sides and across all conditions to ensure comparable responses. Independent of the stimulation amplitude, the SCM response to GVS was always clearly visible and greater than the EMG signal at rest (Figure 3, left panel). For the sham stimulation, a mechanical vibration (400 Hz) was also delivered on the tested side (same location as the positive electrode for GVS). This approach provides a cutaneous sensation produced by the vibration that mimics the tactile input associated with GVS without delivering a stimulating current. As a result, participants are exposed to a similar sensory experience while ensuring that vestibular pathways are not stimulated. Participants from the stroke group completed nine randomized conditions with each arm: three tasks (rest, reaching with neutral and abducted shoulder) and three stimulus types (GVS, none and sham). All participants in the control group performed these tasks using their right arm. To exclude the potential confounding effect of limb dominance in control subjects, we tested for differences within left versus right sides in a subset of participants (*n* = 7). All subsequent comparisons, both within the stroke group and between groups (stroke vs. control), were conducted including all 14 participants per group.

No carryover effects from GVS or sham stimulation have been observed previously (Bartsch & Valero‐Cuevas, 2025); however, the rest + no stimulation condition was always completed first, and all others were randomized.

### 2.5 Data acquisition and processing

A custom game was designed in c# to collect the angle data from the ergometer and provide live real‐time feedback about the rotation velocity of the user (Unity3D, San Francisco, CA, USA). The game featured a dolphin that moved vertically on the screen in real time, with its position directly linked to the rotation speed of the participant (0.5 Hz or 2 s per rotation). When the participant maintained the target speed, the dolphin aligned with a horizontal reference line centred on the display. If the rotation speed decreased, the dolphin sank below the line; if the speed increased beyond the target, the dolphin rose above it. Custom hardware provided a pulse via an Arduino MEGA (Arduino, Somerville, MA, USA) to synchronize EMG data, ergometer angle measurements and delivery time of GVS stimuli. We collected EMG signals at 2.5 kHz from seven muscles of each arm separately using a DataLINK system and associated software (Biometrics Ltd, Newport, UK). Surface EMG sensors (Biometrics Ltd SX230: bipolar, gain 1000, bandwidth 20–460 Hz) were placed over the arm: biceps brachii (Bic), lateral head of the triceps brachii (Tric), anterior, middle and posterior deltoid (ADelt, MDelt and PDelt, respectively), upper trapezius (UTrap) and sternocleidomastoid (SCM) muscles, following standard recommendations from SENIAM (Surface Electromyography for the Non‐Invasive Assessment of Muscles project). Electrode placement and signal quality were confirmed using palpation of each muscle and observation of the EMG during voluntary activation. This set of muscles is sufficient for a general analysis of coupling among the shoulder/elbow muscles relevant to our task (Bartsch & Valero‐Cuevas, 2025; Laine et al., 2021).

To remove GVS artefacts, we inspected the spike‐triggered average of each participant's trial visually to identify the duration of the stimulation artefact. On average, the artefact began 4.8 ms before and ended 8.4 ms after the electrical pulse was triggered. To prevent aliasing and preserve the frequency characteristics of the signal, the artefacts were replaced with interpolated data from the corresponding SCM, deltoid (ADelt, MDelt and PDelt) and UTrap EMG signals (Figure 3, left panel). Given that vestibular responses have a latency of 8–50 ms, we prevented their removal from the signals during the replacing and interpolation process. (Forbes et al., 2015). Signal processing was done according to our previously published papers (for a detailed description, see Bartsch & Valero‐Cuevas, 2025; Laine et al., 2021). In summary, all EMG signals were downsampled to 1000 Hz, bandpass filtered between 8 and 250 Hz, then full‐wave rectified. The purpose of the filter was to remove any remaining artefacts arising from GVS and from those frequencies irrelevant for IMC analysis. EMG signals were full‐wave rectified to improve the detection of synchronized oscillatory neural inputs shared across muscles, while preserving the signal characteristics required for reliable coherence analysis (Boonstra & Breakspear, 2012).

### 2.6 Statistical analysis

IMC assesses shared neural drive between two muscles by measuring the synchronization of the common frequency components in their EMG signals (Boonstra, 2013). As opposed to time‐domain correlation, which measures how two signals co‐vary over time, IMC specifically evaluates correlation in the frequency domain. Consequently, low or negligible IMC is expected at rest, whereas higher IMC is expected when a motor task increases the shared neural drive to the muscles. Therefore, the resting condition is an ideal baseline against which to test whether GVS elicits an increase in shared neural drive to neck or arm muscles. We calculated magnitude squared coherence between each muscle pair using 300 ms windows and a 50% overlap (Bartsch & Valero‐Cuevas, 2025; Laine et al., 2021). A significant pairwise coherence threshold was built for visual inspection. We generated 1000 phase‐randomized surrogate series for each muscle pair and participant following the methods described by Ebisuzaki (1997) (for a detailed description, see Bartsch & Valero‐Cuevas, 2025). Individual pairwise coherence above the 95% confidence interval (CI) is likely to be significant, revealing true shared drive between the two muscles. Averaging correlation coefficients (ρ) leads to biased parameter estimates. Consequently, we applied Fisher's *z*‐transformation [IMC*_z_ *= atanh(ρ)] to all IMC values to ensure unbiased statistical testing. This transformation was done on an individual basis (for each trial) before performing statistical comparisons (Bartsch & Valero‐Cuevas, 2025; Laine et al., 2021). We compared IMC*_z_* using a linear mixed‐effects model with fixed effects of stimulus type (none, GVS and sham), sides (left and right in control subjects or paretic and non‐paretic in stroke survivors) and band (alpha, 8–16 Hz; beta, 16–30 Hz; gamma, 30–50 Hz), with subject as a random effect. Consequently, our design allowed us to test for within‐subject differences (e.g., left vs. right or paretic vs. non‐paretic) and between‐subject contrasts (e.g., control–right vs. stroke–paretic). The values of IMC*_z_* across the frequency spectrum were gathered into three bands for two reasons. First, these bands are thought to reflect the origin of shared neural drive; beta‐band coherence has been associated with CST activity and is considered a marker of CST dysfunction (Fisher et al., 2012), whereas alpha and alpha‐to‐gamma bands are thought to reflect reticulospinal (Grosse & Brown, 2003) and vestibulospinal pathways, respectively. Second, aggregating coherence values into broader frequency bands helps to reduce the number of *post hoc* comparisons, thereby lowering the risk of type I error and increasing the robustness of statistical inferences when evaluating group or task‐related differences. Given violations of model assumptions and the lack of a non‐parametric alternative for testing our hypotheses, we used a robust ANOVA model to assess whether stroke and GVS increases shared neural drive. The advantage of these robust methods is that they provide unbiased parametric estimates, even when data depart from normality or when statistical assumptions are not met fully. *Post hoc* comparisons were conducted with Bonferroni corrections when more than two conditions were compared (e.g., stimulus type). For comparisons involving only two conditions (e.g., side comparisons), no further analysis was performed because it was deemed unnecessary. All signal processing procedures and statistical analyses were performed using R/Rstudio and the corresponing robust statistical packages (Lenth, 2025; Mair & Wilcox, 2020; R Core Team, 2021).

Because our experimental design involved testing for multiple main effects (task, stimulus type, group and laterality), we implemented a stepwise statistical approach to minimize the number of comparisons and maximize statistical power. First, the rest condition was used to confirm the expected GVS‐evoked responses in neck muscles and for testing whether IMC to arm muscles increases during GVS. Therefore, no direct comparisons were performed between rest and reaching tasks. Second, comparisons of left and right sides were conducted only in the subset of control participants (*n* = 7). After ruling out dominance‐related effects, data from the left side of controls were excluded from further analyses. All subsequent comparisons involved the right side of control subjects, which was compared with each side of participants with stroke. Third, the analyses of stimulus type were structured to reduce unnecessary contrasts. After identifying a main effect of stimulus type but no effect of sham stimulation, data from the sham condition were removed from subsequent analyses. Likewise, because GVS did not change intermuscular coherence in arm muscles (at rest or during reaching tasks), GVS trials were excluded from the remaining task‐related analyses. Only when a main effect of stimulus type was found, we performed *post hoc* analyses to determine the frequency bands in which these differences occurred. Finally, after these data‐reduction steps, the primary comparisons focused on: (1) the effect of GVS on neck muscles from both sides of stroke during rest; and (2) during reaching, the effects of task (neutral vs. abducted shoulder) and group (control–right side vs. stroke–paretic side). This multistep analytical strategy ensured that each level of analysis was guided by preceding results, thereby reducing the total number of comparisons and preserving statistical power while accurately reflecting the hierarchical structure of the experimental design.

## 3 RESULTS

### 3.1 GVS increases neural drive in neck muscles on both sides of the control group at rest

Initially, we compared shared neural drive, as measured by IMC*_z_*, on both sides of the control group (neurotypical participants) to exclude arm dominance as a confounder and to compare the right side of control subjects with both the non‐paretic and paretic arms of stroke survivors. The mean IMC*_z_* (across all frequencies) between both sides (right vs. left) during rest in a subset of participants (*n* = 7) showed no significant differences as a function of side in any muscle pair (SCM–UTrap, *P* = 0.28; Bic–ADelt, *P* = 0.49; Bic–MDel, *P* = 0.66; and Bic–PDel, *P* = 0.39).

As expected, we did find statistical differences in shared neural drive to neck muscles attributable to stimulus type (SCM–UTrap, *P* = 0.01; Tables 2 and 3). After confirming that the type of stimulation had a significant overall effect on IMC*_z_* for neck muscles (SCM–UTrap), we conducted *post hoc* analyses to identify which type of stimulus (i.e., no stimulation, GVS or sham) and which frequency bands (alpha, beta or gamma) showed statistically significant IMC*_z_* changes. The ANOVA results confirmed previous findings in younger control participants: (1) shared neural drive between neck muscles at rest increased only during GVS (alpha, *P* = 0.002; beta, *P* *<* 0.001; and gamma, *P* *<* 0.001); and (2) no significant differences were found in IMC*_z_* for neck muscles (SCM–UTrap) between no stimulation and sham stimulus types (*P* = 1 across frequency bands owing to Bonferroni corrections). These results confirm the notion that GVS increases brainstem vestibular output only to neck muscles in the present older control group, as previously found in young neurotypical participants (Bartsch & Valero‐Cuevas, 2025).

### 3.2 GVS does not increase common neural drive to neck muscles at rest in the non‐paretic side after stroke

The same comparisons between sides of the body were performed in the stroke group to identify changes in IMC*_z_* between both sides and stimulus types during rest. Similar to the control group, stroke survivors showed statistical differences between stimulus types (*P* = 0.001). As opposed to control subjects, however, we did find statistical differences between sides (paretic vs. non‐paretic) in neck muscles (SCM–UTrap, *P* = 0.037; Table 3), in addition to an interaction (stimulus type *×* side, *P* = 0.031; Table 3). Consequently, stroke (but not control) participants had differences between sides, probably as a result of the hemiparetic stroke, because dominance was excluded as a confounder in the control group.

Given that no changes in IMC*_z_* were found between the right and left sides in control participants, we selected the right side as the reference for subsequent comparisons with the stroke group, who were selected for having paresis in their right, previously dominant, arm. To assess whether each side of the stroke participants differed from the control group, we performed separate robust ANOVAs comparing control participants (right side) with the paretic and non‐paretic sides of stroke survivors (main effects for group and stimulus type for each muscle pair). Unexpectedly, significant group differences were found at rest, but only when comparing control participants with the non‐paretic side (*P* = 0.031), not with the paretic side of stroke participants (*P* = 0.428; Tables A1 and A2). Regarding stimulus type, we found an overall effect on neck muscle IMC*_z_* when comparing each side of stroke participants with control participants (Table A1). More specifically, when GVS was applied at rest, Bonferroni‐corrected *post hoc* analyses revealed significantly lower alpha‐band IMC*_z_* in neck muscles from the non‐paretic side in comparison to control participants (*P* = 0.029; Figure 3). In contrast, also when GVS was applied at rest on the same side of the body, no differences in IMC*_z_* were found between neck muscles from the paretic side and control participants (*P* = 0.38). These differences in shared neural drive to neck muscles within the paretic and non‐paretic sides of stroke survivors were further supported after comparing each side of stroke participants with control participants. Importantly, these differences were driven primarily by a reduction in neural drive on the non‐paretic (ipsilesional) side, rather than by upregulated drive on the paretic (contralesional) side, when GVS was applied at rest.

**TABLE 1:** Group summary and per‐subject (stroke) demographics and clinical characteristics for participants in the control and stroke groups.

Group	*n*	Mean (±SD) age (years)	Sex (male/female)	Mean Fugl‐Meyer score (±SD)	Stroke onset mean (range) (years)
Stroke	14	58.5 (±8.0)	9/5	4.6 (1.1–14.1)	4.6 (1.1–14.1)
Participant	1	70	Male	60	6.3
	2^a^	59	Male	6	2.5
	3	53	Male	57	6.5
	4	50	Male	43	14.1
	5^b^	49	Male	50	3.4
	6	54	Female	48	5.8
	7	67	Female	51	2.6
	8	75	Female	29	1.4
	9	57	Male	58	2.1
	10	52	Female	60	1.1
	11	55	Male	58	9.7
	12	66	Male	28	1.1
	13^a^	54	Female	6	4
	14	58	Male	19	3.8
Control	14	58.3 (±8.6)	10/4	–	–

^a^ Participants unable to perform motor tasks with abducted shoulder.

^b^ Participant unable to complete all tasks due to technical difficulties.

**TABLE 2:** Summary of results comparing intermuscular coherence in neck and arm muscles between control participants and/or both sides of the body in stroke survivors with hemiparesis.

Group (side)	GVS at rest	GVS during reach	Neutral vs. abducted
Control (R) vs. stroke (P)	Neck: control ≈ P (T4, F3)	Control ≈ P^a^	Arm: control > paretic (T5)
Control (R) vs. stroke (NP)	Neck: control > NP (T4, F3)	–^b^	–
Stroke (P vs. NP)	Neck: *P* > NP (T3)	–^b^	–

*Note*: Sides: right dominant paretic (P); left non‐dominant non‐paretic (NP); and dominant right (R). Neck muscles: sternocleidomastoid (SCM) and upper trapezius (UTrap). Arm muscles: biceps (Bic) and delt (anterior, middle and posterior). See Results for details or references included for tables (T) and figures (F).

^a^ Galvanic vestibular stimulation (GVS) did not increase coherence between any neck or arm muscle pair.

^b^ Coherence between neck and arm muscles did not differ between control subjects and the paretic side of stroke survivors, consistent with previous findings in young neurotypical adults (Bartsch & Valero‐Cuevas, 2025).

No comparisons were made within stroke sides or between the control (R) and the non‐paretic (NP) side.

### 3.3 Decreased neural drive to paretic arm muscles when reaching with an abducted shoulder after stroke

During the reaching tasks, we first compared the angular velocity of the movement to confirm that motor performance was comparable between groups. This ensured that any observed differences in shared neural drive following stroke could be attributed to changes in neural drive rather than performance‐related factors. The right arm of control subjects and the paretic arm of stroke survivors showed average velocities of 0.593 and 0.574 cycles s^−1^, respectively. ANOVA comparison between group (control vs. stroke) and task (reaching with neutral vs. abducted shoulder) revealed no significant differences in angular velocity between the right arm of control subjects and the paretic arm of stroke survivors, nor between reaching tasks (task, *P* = 0.42; group, *P* = 0.09; task × group, *P* = 0.30).

We assessed the effect of stimulus type on shared neural drive during reaching in stroke survivors. The results revealed that GVS did not increase IMC*_z_* to neck muscles (SCM–UTrap) for the paretic side of stroke survivors. This finding is supported by the ANOVA in both neutral (*P* = 0.142) and abducted shoulder positions (*P* = 0.857). Likewise, GVS did not increase neural drive to any arm muscle on the paretic side (neutral/abducted: Bic–ADelt, *P* = 0.31/0.73; Bic–MDelt, *P* = 0.71/0.9; Bic–PDelt, *P* = 0.45/0.97). Importantly, these results provide evidence that excludes a vestibular contribution to upper arm neural drive during unperturbed voluntary reaching movements.

After we confirmed that GVS or sham did not change IMC during the reaching tasks, we removed these conditions from further analysis.

**FIGURE 4:**
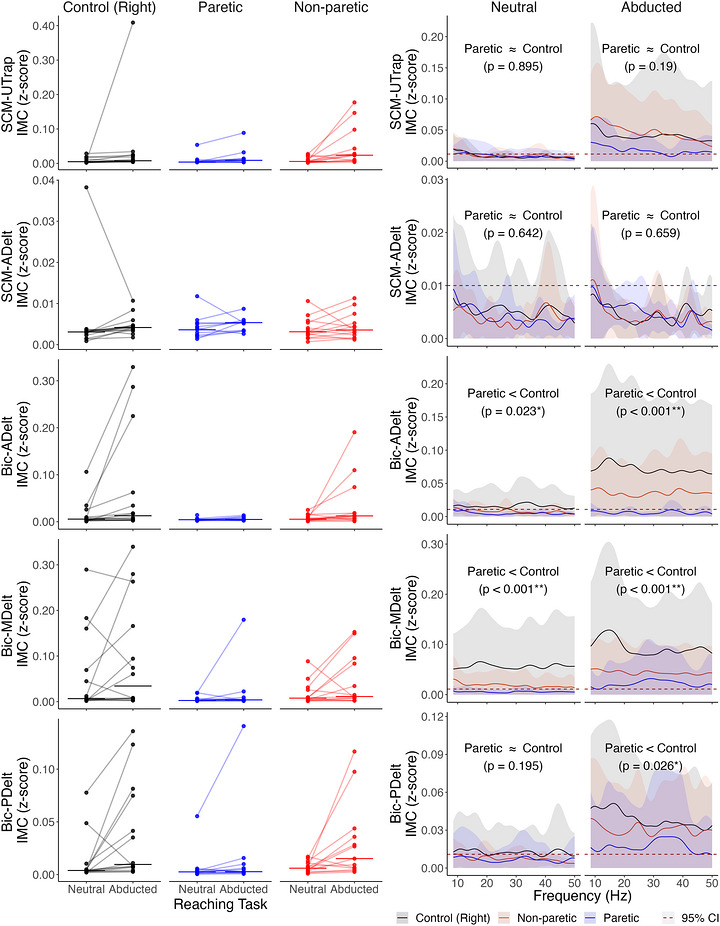
The paretic side of stroke survivors had lower intermuscular coherence *z*‐score (IMC*_z_*) when compared with control participants. Left panels, mean IMC*_z_* for each participant during both reaching tasks. Right panels, group mean IMC*_z_* for control participants and each side of stroke participants. Abbreviations: ADelt, anterior deltoid; Bic, biceps brachii; MDelt, middle deltoid; PDelt, posterior deltoid; SCM, Sternocleidomastoid; UTrap, upper trapezius.

During reaching, we found that the paretic side of stroke survivors showed significantly lower coherence when compared with control participants. This is evidenced by the overall group differences between biceps and deltoid (Bic–ADelt, *P* = 0.005; Bic–MDelt, *P* = 0.004; Bic–PDelt, *P* = 0.048; ADelt–MDelt, *P* *<* 0.001; Table A2). Likewise, the reaching tasks also showed lower IMC between neutral and abducted positions of the shoulder (Bic–ADelt, *P* = 0.007; Bic–MDelt, *P* = 0.005; Bic–PDelt, *P* = 0.003; ADelt–MDelt, *P* = 0.963; Table A2). Finally, a detailed analysis found that the Bic–ADelt and Bic–MDelt muscles from the paretic side had lower coherence in both reaching tasks when compared with control participants (neutral/abducted: Bic–ADelt, *P* = 0.023/0.001; Bic–MDelt, *P* = 0.001/0.001; Figure 4). The Bic–PDelt muscle pair in the paretic side had lower coherence when reaching with abducted shoulder only (*P* = 0.026), when compared with control participants.

## 4 DISCUSSION

Our findings confirm that, in neurotypical participants (control group) at rest, neck muscles receive shared neural drive, as measured by IMC*_z_*, from the vestibular system (i.e., brainstem vestibular output). Specifically, when GVS is applied at rest, the baseline shared neural drive increases in a similar manner on both sides, indicating no lateral dominance. Supporting these results, we observed no increase in IMC*_z_* during sham when compared with no stimulation, excluding a proprioceptive or placebo effect as a likely mechanism for the GVS‐driven changes. Moreover, they also confirm the absence of IMC*_z_*, or lack of increase with GVS, between arm muscles (Bic–ADelt, Bic–MDelt and Bic–PDelt) when reaching. This suggests that, in stroke survivors, arm muscles are unlikely to receive increased brainstem vestibular output during either rest or unperturbed reaching.

### 4.1 Decreased neural drive to neck muscles is limited to the non‐paretic (ipsilesional) side when GVS is applied at rest

In contrast to the control subjects (and neurotypical younger adults; Bartsch & Valero‐Cuevas, 2025), stroke survivors exhibited a reduction in shared vestibular drive to the non‐paretic (ipsilesional) side of neck muscles at rest (Figure 3; Table 3). This asymmetry is consistent with previous reports of vestibular‐evoked myogenic potentials in the SCM muscle following stroke. Specifically, the ratio of the muscle peak amplitude responses between the paretic and non‐paretic sides favoured the amplitudes of the paretic side (Miller et al., 2014). However, our detailed comparison between control and both sides of stroke survivors revealed that these asymmetries arise from decreased GVS responses on the non‐paretic side rather than increased responses on the paretic side. In fact, our results also revealed lower amplitudes in muscle responses to GVS in the SCM and the UTrap muscles from the non‐paretic side compared with both the paretic side and control participants (Figure 3). This finding supports various forms of disruption to ipsilesional neural drive to the neck muscles. Furthermore, given that the vestibular nuclei also project in an ascending way to cortical areas (Fukushima, 1997) and that vestibular stimulation preferentially activates the ipsilateral hemisphere (Indovina et al., 2020; Lopez et al., 2012; Schlindwein et al., 2008), the selective decrease in coherence between neck muscles on the non‐paretic side is likely to reflect disruptions or downregulation of ipsilesional cortico‐vestibular pathways in the stroke group. This interpretation is supported by marked loss of head stabilization reflexes following stroke and vestibular dysfunction. The head stabilization reflexes rely on excitatory inputs from both cortical and vestibular structures, which are thought to reduce excitatory drive to ipsilesional neck muscles (Bademkiran et al., 2016). Likewise, the ipsilesional arm of stroke survivors exhibits hemisphere‐specific motor deficits, with the dominant hemisphere primarily affecting dynamic control and the non‐dominant hemisphere steady‐state control (Mani et al., 2013; Schaefer et al., 2007). These deficits, which also include impaired coordination, reduced gross and fine motor dexterity, and generalized muscle weakness across both upper and lower limbs, might contribute further to the reduced ipsilesional coherence in neck muscles observed in our study (Bustrén et al., 2017; Pandian & Arya, 2013; Son et al., 2018).

**TABLE 3:** At rest, the stroke group showed the same baseline coherence and its increase with galvanic vestibular stimulation in neck muscles as neurotypical younger adults (Bartsch & Valero‐Cuevas, 2025) and the age‐matched control subjects, but only on their paretic (contralesional) side.

Muscle pair	Group	Effect (factors)	*F* ratio	DF_1_	DF_2_	*P*‐value
SCM–UTrap	Control (*n* = 7)	Side (L/R)	1.26	1.00	11.90	0.28
		Stimulus type (none, sham, GVS)	7.72	2.00	10.00	0.01**
		Stimulus type *×* side	0.27	2.00	10.00	0.77
	Stroke (*n* = 14)	Side (paretic/non‐paretic)	4.97	1.00	20.10	0.037*
		Stimulus type	15.72	1.00	22.40	0.001**
		Stimulus type *×* side	5.29	1.00	22.40	0.031*

*Note*: Significant intermuscular coherence (IMC*_z_*) differences between the paretic and non‐paretic sides in neck muscles (sternocleidomastoid and upper trapezius; SCM–UTrap). Both groups showed IMC*_z_* differences attributable to stimulus type, also in neck muscles. Red text indicates significant differences at *0.05 and **0.01 levels.

Abbreviations: GVS, galvanic vestibular stimulation; L, left, R, right.

Finally, increased alpha band IMC was consistently shown in both sides of control participants and the paretic side of stroke survivors, in addition to young neurotypical subjects (Bartsch & Valero‐Cuevas, 2025). Previous evidence has shown a similar increase in brainstem vestibular output (Blouin et al., 2011; Forbes et al., 2015), which decreases during voluntary arm movement (Bartsch & Valero‐Cuevas, 2025; Malone et al., 2025). In contrast, the non‐paretic side of stroke survivors showed decreased IMC to neck muscles when GVS was applied at rest. This selective reduction provides evidence for an ipsilateral vestibular origin for the disrupted neural drive to neck muscles, which needs to be addressed further.

### 4.2 Decreased neural drive to paretic arm muscles when reaching

Following upper motor neuron damage, a decrease in shared neural drive has been reported during voluntary movements, such as reaching, precision grip and pinching tasks. This reduction in IMC has been observed across frequency bands (Fang et al., 2009; Fisher et al., 2012; Larsen et al., 2017). For example, IMC in the beta band (16–30 Hz) was found to be decreased in patients with corticospinal tract degeneration (as seen in primary lateral sclerosis), whereas it remained intact in patients with lower motor neuron disease (as seen in progressive muscular atrophy) (Fisher et al., 2012). Likewise, stroke survivors have shown lower alpha and beta band coherence in hand muscles (first dorsal interosseous muscle, adductor pollicis and abductor pollicis brevis) during pinching tasks (Larsen et al., 2017), in addition to gamma band corticomuscular coherence in arm muscles (anterior deltoid, biceps and triceps brachii) during reaching tasks (Fang et al., 2009). In agreement with these findings, during voluntary reaching (neutral and abducted shoulder positions), the paretic side of stroke survivors showed a decrease in shared neural drive to biceps and deltoid muscles in comparison to control participants (Figure 4; Table A2). Given our new finding of a lack of increased coherence when GVS was applied during the reaching movements, it is likely that this observed decrease in coherence in arm muscles arises from the disruptions in CST drive following stroke, rather than disruptions in brainstem vestibular output. Our protocol did not include measurement of CST integrity to test this hypothesis. However, this is supported by previous evidence, in which coherence has been shown to decrease in upper but not lower motor neuron injury (Fisher et al., 2012), and during pharmacological inhibition of somatosensory areas in the cortex of healthy participants (Baker & Baker, 2003).

Although our study has limitations, we believe they do not undermine our primary findings. For instance, increased coherence in neck muscles in stroke survivors owing to GVS might be attributed to direct electrical stimulation of the muscles or activation of the accessory (XI) cranial nerve, given the location and proximity of the GVS and EMG electrodes. However, such responses have been observed consistently in both our control group and young neurotypical participants (Bartsch & Valero‐Cuevas, 2025). Notably, these responses occur with a longer latency than would be expected from direct stimulation alone (as seen in Figure 3). Furthermore, the decreased coherence observed in neck muscles of the non‐paretic side of stroke survivors and its suppression during voluntary movement in control participants would not occur if direct stimulation were solely responsible. Lastly, our artefact removal process, applied after each stimulation pulse, further minimizes the influence of direct stimulation (Pinto & De Carvalho, 2008). This process, however, might also lead to an artefactual increase in coherence below 8 Hz and its 4 Hz harmonics. Likewise, vagus nerve stimulation (VNS) is a potential confounder for the increased coherence we see between neck muscles. Transcutaneous auricular VNS (taVNS) is delivered using surface electrodes placed on vagally innervated regions of the ear, typically with both electrodes positioned on the tragus (Farmer et al., 2021). However, this configuration differs fundamentally from GVS used in our work, where the electrodes are placed bilaterally on the mastoid processes. Because taVNS and GVS activate anatomically distinct structures with clearly separated electrode locations, GVS is unlikely to produce simultaneous stimulation of the vestibular and vagus nerves. Moreover, the therapeutic effects of VNS in stroke have been shown to reduce brain damage by modulating anti‐inflammatory and neuroprotective pathways. In addition, VNS appears to improve motor recovery primarily through sensory‐driven (rather than motor) mechanisms (Adair et al., 2020; Baig et al., 2022). Therefore, we conclude that VNS is most likely not to be the operant mechanism that produced the coherence results in this study.

When considering the decreased drive to neck muscles on the non‐paretic side, one alternative explanation is an abnormal reliance on ipsilateral innervation, rather than the widely accepted contralateral innervation to muscles (Natali et al., 2018; Porter & Lemon, 1995). For instance, it is known that ipsilateral axial muscles receive input from 5%–15% of undecussated corticospinal fibres (Natali et al., 2018; Porter & Lemon, 1995). Likewise, the SCM muscle, which consists of two differentially innervated muscle bellies which cannot be distinguished using surface EMG electrodes, exhibits ipsilateral innervation (Balagura & Katz, 1980; DeToledo & David, 2001; Mazzini & Schieppati, 1992), and clinical studies have reported SCM weakness on the non‐paretic side following stroke. The upper trapezius, in contrast, shows weakness on the paretic side (Anagnostou et al., 2011; Manon‐Espaillat & Ruff, 1988). Despite these observations, we consider this alternative explanation (ipsilateral innervation) unlikely because: (1) at most, 5%–15% of the corticospinal tract remains undecussated; (2) ipsilateral innervation has been documented for the SCM only; and (3) our results clearly show lower responses in both the SCM and upper trapezius muscles, of which only the latter is likely to be innervated by the non‐lesioned hemisphere. Given these inconsistencies, we propose that disruptions in ipsilateral cortico‐vestibular pathways are the most plausible explanation for the observed decrease in shared neural drive to neck muscle of the non‐paretic side in stroke.

Another limitation of this study is the relatively small sample size and wide range of severity of impairment across participants. The sample size per group (*n* = 14), although modest, was guided by prior IMC and GVS studies demonstrating robust effect sizes in similar populations (Bartsch & Valero‐Cuevas, 2025; Laine et al., 2021). From a statistical perspective, the within‐subject repeated‐measures design increased statistical power by allowing each participant to serve as their own control. Additionally, robust statistical methods were used throughout, relaxing standard parametric assumptions and mitigating concerns about sample size further. Importantly, the primary GVS finding is a null result, which is less susceptible to a false positive than significant findings from an insufficient sample size. The adequacy of the design is supported by its sensitivity to detect the expected paretic–non‐paretic differences in IMC in the stroke group and the expected absence of left–right differences in control subjects. Regarding motor impairment severity and their impact on our results, it has been shown that shoulder abduction reliably exacerbates expression of pathological synergy even in mild‐to‐moderate stroke (Dewald et al., 1995; McPherson et al., 2018). Consistent with this, the EMG data confirmed increased biceps and deltoid activity during abduction in the paretic arm (Figure 2). Nonetheless, it remains possible that vestibular contributions to expression of synergy differ across severity levels. Specifically, participants with limited CST damage might not exhibit vestibular upregulation, potentially explaining the absence of GVS‐driven changes in IMC in paretic arm muscles. Future studies with larger, severity‐stratified cohorts are needed to test whether vestibular contributions become more prominent as impairment severity increases and CST damage is more severe. Finally, given that we did not perform a topographic or neuroanatomical analysis of stroke‐related neural damage in these stroke survivors, particularly within cortico‐vestibular and brainstem pathways, it remains possible that some of the decreased neural drive on the paretic side could reflect dysregulation of uninjured pathways. Nonetheless, our findings agree with previous reports on stroke and upper motor neuron injuries, which strongly suggest that decreased neural drive results primarily from damage to motor pathways following stroke.

Our findings carry specific implications for neurorehabilitation and neuromodulation protocols, even in the absence of a GVS‐driven effect on the paretic arm of stroke survivors. First, the absence of increased vestibular drive during GVS is itself clinically informative; it argues against vestibulospinal dysregulation as a meaningful therapeutic target for reducing pathological synergies and, consequently, reinforces the focus on residual corticospinal and brainstem projections as primary drivers of motor impairment. Moreover, it highlights the need to consider other circuits that might contribute to pathological synergies and altered muscle tone, including propriospinal relay neurons, spinal interneuron circuits such as Ia inhibitory interneurons and Renshaw cells, and monoaminergic descending pathways known to modulate motor neuron excitability. These represent underexplored but potentially important targets for future neuromodulatory and rehabilitative strategies aimed at restoring voluntary arm control after stroke. Second, the selective decrease in vestibular drive to neck muscles on the non‐paretic (ipsilesional) side reveals that ipsilesional brainstem pathways are disrupted following stroke. This strengthens the growing notion that stroke affects both sides of the body, further discouraging the use of the terms ‘non‐affected/healthy’ for the ipsilesional hemibody and highlighting the need to include this often‐overlooked side in both assessment and treatment. This has direct relevance for neuromodulatory strategies, such as non‐invasive brain stimulation or GVS‐based protocols, targeting ipsilesional cortical and brainstem circuits, with the goal of restoring, rather than suppressing, vestibular drive to neck and axial muscles on the ipsilesional side. Finally, coherence in paretic arm muscles was reduced further when the shoulder was abducted, which is the same posture that exacerbates flexion synergies in stroke. This suggests that the effectiveness of neuromodulation protocols might depend on the arm position used during treatment, which future studies should consider carefully.

## 5 CONCLUSION

In summary, our study confirms that GVS produces a reliable increase in the shared neural drive (IMC*_z_*) to neck muscles of neurotypical individuals, as seen in our age‐matched control group and younger neurotypical participants (Bartsch & Valero‐Cuevas, 2025), at rest. In contrast, stroke survivors exhibit an asymmetric response; the paretic side shows increased GVS‐induced shared neural drive to neck muscles comparable to control participants, whereas the non‐paretic (ipsilesional) side is unexpectedly decreased. This decreased neural drive suggests a disruption of ipsilesional cortico‐vestibular and brainstem pathways. Furthermore, our results in stroke survivors reveal that shared neural drive in arm muscles is particularly decreased during reaching with an abducted shoulder (the posture that exacerbates arm flexion synergies in stroke). However, because GVS did not increase shared neural drive during reaching movements (with or without GVS), our findings exclude a significant contribution from brainstem vestibular output to arm motor impairment, instead highlighting the likely role of the remaining pathways to the disruption of voluntary arm movements following stroke.

## AUTHOR CONTRIBUTIONS

Angelo Bartsch‐Jimenez and Francisco J. Valero‐Cuevas contributed to conception and design of the study. Angelo Bartsch‐Jimenez and Hesam Azadjou performed the experiments, preprocessed the data and performed the statistical analysis. Angelo Bartsch‐Jimenez, Hesam Azadjou and Francisco J. Valero‐Cuevas interpreted the results and wrote the manuscript. All authors contributed to manuscript revision, approved the final version of the manuscript and agree to be accountable for all aspects of the work in ensuring that questions related to the accuracy or integrity of any part of the work are appropriately investigated and resolved. All persons designated as authors qualify for authorship, and all those who qualify for authorship are listed.

## CONFLICT OF INTEREST

The authors declare that the research was conducted in the absence of any commercial or financial relationships that could be construed as a potential conflict of interest.

## Data Availability

The data that support the findings of this study are available from the corresponding author upon reasonable request.

## 1

**TABLE A1:** When galvanic vestibular stimulation is applied at rest, the non‐paretic side of stroke patients showed significant lower levels of alpha‐band IMC*_z_* in neck muscles when compared with control participants.

Group	Band	Muscle	Effect	Statistic		*P*‐value	
				Paretic	Non‐paretic	Paretic	Non‐paretic
		alpha	Group	0.65	5.38	0.428	**0.031***
			Stimulus type	16.52	6.62	**0.001***	**0.018***
	SCM–UTrap		Group *×* stimulus type	0.05	2.63	0.82	0.121
		beta	Group	0.07	2.41	0.802	0.137
			Stimulus type	21.01	12.28	**0.001***	**0.002***
			Group *×* stimulus type	0.01	1.72	0.914	0.205
		gamma	Group	0.17	2.59	0.688	0.126
			Stimulus type	12.85	6.00	**0.002***	**0.025***
			Group *×* stimulus type	0.01	1.91	0.942	0.185
		alpha	Group	0.76	0.04	0.396	0.846
			Stimulus type	2.43	1.03	0.136	0.319
	Bic–ADelt		Group *×* stimulus type	0.61	0.72	0.446	0.402
Control vs. stroke		beta	Group	0.27	0.11	0.61	0.745
			Stimulus type	5.96	3.98	**0.022***	0.057
			Group *×* stimulus type	0.14	0.03	0.716	0.875
		gamma	Group	0.44	0.43	0.516	0.51
			Stimulus type	3.56	0.89	0.076	0.354
			Group *×* stimulus type	0.48	1.26	0.499	0.27
		alpha	Group	1.09	0.01	0.31	0.913
			Stimulus type	2.23	1.08	0.152	0.307
			Group *×* stimulus type	0.08	0.12	0.786	0.729
	Bic–MDelt	beta					
			Group	0.56	0.01	0.46	0.942
			Stimulus type	4.34	2.94	**0.046***	0.098
			Group *×* stimulus type	0.01	0.04	0.906	0.844
		gamma	Group	0.45	0.15	0.509	0.702
			Stimulus type	3.09	1.29	0.094	0.265
			Group *×* stimulus type	0.09	0.26	0.772	0.616
		alpha	Group	0.61	0.09	0.445	0.767
			Stimulus type	2.73	0.80	0.117	0.381
	Bic–PDelt		Group *×* stimulus type	0.22	1.27	0.647	0.274
		beta	Group	0.09	0.04	0.77	0.852
			Stimulus type	6.46	4.12	**0.018***	0.053
			Group *×* stimulus type	0.00	0.13	0.994	0.718
		gamma	Group	0.28	0.04	0.601	0.839
			Stimulus type	3.66	1.59	0.073	0.219
			Group *×* stimulus type	0.23	0.77	0.638	0.39

*Note*: Bold red text indicates significant differences at *0.05 and **0.01 levels.

Abbreviations: ADelt, anterior deltoid; Bic, biceps brachii; IMC, intermuscular coherence; MDelt, middle deltoid; PDelt, posterior deltoid; SCM, Sternocleidomastoid; UTrap, upper trapezius.

**TABLE A2:** In comparison to control subjects, the paretic side of stroke participants showed decreased IMC*_z_* to biceps and deltoid during reaching, and for the abducted shoulder position, the paretic side also showed a significantly decreased IMC*_z_* when compared with control participants.

Group	Muscle	Effect	Statistic	*P*‐value
	Bic–ADelt	Shoulder	8.23	**0.007***
		Group	8.82	**0.005***
		Shoulder *×* group	7.36	**0.01***
	Bic–ADelt	Shoulder	8.93	**0.005***
Control vs. paretic side		Group	9.11	**0.004***
		Shoulder *×* group	3.38	0.073
	Bic–MDelt	Shoulder *×* group	3.38	0.073
		Group	4.12	**0.048***
		Shoulder *×* group	2.11	0.153
	ADelt–MDelt	Shoulder	0.00	0.963
		Group	14.09	*<* **0.001***
		Shoulder *×* group	1.03	0.315

*Note*: Bold red text indicates significant differences at *0.05 and **0.01 levels.

Abbreviations: ADelt, anterior deltoid; Bic, biceps brachii; IMC, intermuscular coherence; MDelt, middle deltoid; PDelt, posterior deltoid.
